# Impact of visceral fat area on surgical difficulty during robotic distal pancreatectomy (TAKUMI-2)

**DOI:** 10.1007/s00464-025-11696-3

**Published:** 2025-04-04

**Authors:** Kosei Takagi, Motohiko Yamada, Tomokazu Fuji, Kazuya Yasui, Takeyoshi Nishiyama, Yasuo Nagai, Noriyuki Kanehira, Toshiyoshi Fujiwara

**Affiliations:** Department of Gastroenterological Surgery, Okayama University Graduate School of Medicine, Dentistry, and Pharmaceutical Sciences, 2-5-1 Shikata-cho, Kita-ku, Okayama, 700-8558 Japan

**Keywords:** Robotic distal pancreatectomy, Difficulty score, Visceral fat area

## Abstract

**Background:**

Difficulty scoring systems (DSS) have been developed to quantify the surgical complexity of laparoscopic distal pancreatectomy (LDP). However, few studies have validated these systems in the context of robotic distal pancreatectomy (RDP). Moreover, the impact of body composition on RDP outcomes remains unexplored. This study aimed to investigate the risk factors of surgical difficulty in RDP, including body composition.

**Methods:**

This retrospective study included 72 consecutive patients who underwent RDP at our institution between April 2021 and October 2024. Using a modified DSS for LDP, patients were divided into three difficulty index groups. The association between the difficulty index and outcomes was investigated. Multivariate analyses were performed to identify risk factors associated with surgical difficulty (prolonged operative time) in RDP.

**Results:**

Patients were classified into three difficulty index groups: low (n = 28), intermediate (n = 25), and high (n = 19). Operative time was significantly associated with the surgical index (*P* = 0.01). Moreover, visceral fat area (VFA) was significantly correlated with operative time (r^2^ = 0.10, *P* = 0.008). The multivariate analyses found that VFA (≥ 100 cm^2^) (odds ratio [OR] 5.03, 95% confidence interval [CI] 1.32–22.4, *P* = 0.02), malignancy (OR 4.92, 95% CI 1.50–18.9, *P* = 0.01), and pancreatic resection on the portal vein (OR 4.14, 95% CI 1.24–15.9, *P* = 0.02) were significant risk factors associated with surgical difficulty.

**Conclusion:**

VFA could be a novel and useful factor for assessing the surgical difficulty associated with RDP.

**Supplementary Information:**

The online version contains supplementary material available at 10.1007/s00464-025-11696-3.

Minimally invasive pancreatic surgery has become popular worldwide over the past few decades, supported by increasing evidence [1, 2]. As the use of minimally invasive distal pancreatectomy has increased, the use of robotic approaches for distal pancreatectomy has also risen [3]. Moreover, the feasibility and safety of robotic distal pancreatectomy (RDP) compared with laparoscopic distal pancreatectomy (LDP) have been demonstrated [4]. Although formal training is recommended for the safe implementation of minimally invasive pancreatic surgery, there are no standardized definitions of the parameters to target during the learning curve or how different learning phases should be evaluated [5].

Difficulty scoring systems (DSS) have been developed to quantify the degree of difficulty of the LDP [6, 7]. The original DSS was developed to stratify the surgical complexity of LDP in Japan [6]. Thereafter, the DSS was revised with the introduction of additional parameters such as malignancy and neoadjuvant therapy [7]. DSS may be useful for the appropriate evaluation of surgical difficulty and patient selection for LDP. However, the efficacy of DSS has not yet been validated for RDP. Moreover, few studies have investigated factors associated with surgical difficulty in patients undergoing RDP. In clinical practice, the difficulty of RDP depends on patient and oncological factors. Given the complexity of some patients, especially those with obesity, we hypothesized that body composition may affect the surgical difficulty associated with RDP.

This study, called the Training program in Okayama University for minimally invasive surgery (TAKUMI-2), aimed to investigate risk factors associated with surgical difficulty in RDP and explore the impact of body composition on these challenges.

## Materials and methods

### Study design

This study was approved by the Ethics Committee of Okayama University Hospital (Approval No. 2502-019) and performed in accordance with the tenets of the Declaration of Helsinki. The need for written informed consent was waived because of the retrospective nature of the analysis of anonymized clinical data. This single-center retrospective study included 72 consecutive patients who underwent RDP at our institution between April 2021 and October 2024.

### Clinical data

Using a prospectively collected database, the following data were extracted: age, sex, American Society of Anesthesiologists physical status, comorbidities (diabetes and hypertension), primary disease (pancreatic cancer, neuroendocrine neoplasm, intraductal papillary mucinous neoplasm, mucinous cystic neoplasm, and others), body composition (body mass index, subcutaneous fat area [SFA], and visceral fat area [VFA]), difficulty parameters [7], operative outcomes (operative time, estimated blood loss, and conversion to open surgery), and postoperative outcomes (mortality, major complication [grade ≥ 3] [8], postoperative pancreatic fistula [POPF; ≥ grade B] [9], postpancreatectomy hemorrhage [PPH], hospital stay, and readmission). Complications within 1 month of surgery were recorded and evaluated. A textbook outcome was defined as the absence of mortality, major complications, POPF, PPH, and readmission [10].

### Image analysis of body composition

**Fig. 1:**
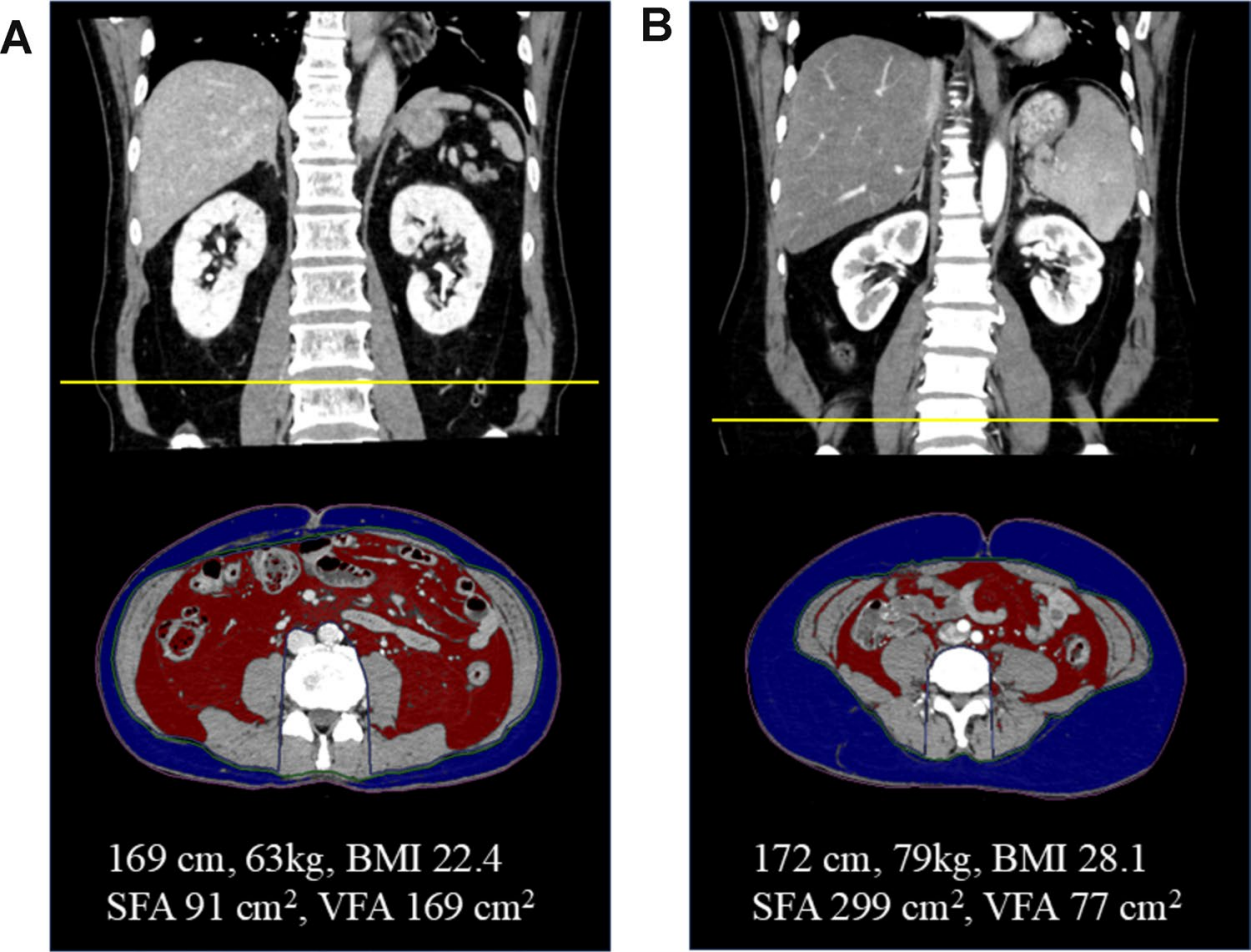
Image analysis of body composition. The subcutaneous fat area (SFA) and visceral fat area (VFA) are calculated using the Synapse Vincent. **A** A patient measuring 169 cm in height and weighing 63 kg. **B** A patient measuring 172 cm in height and weighing 79 kg

Preoperative diagnostic computed tomography images obtained within 2 months before surgery were analyzed to calculate the SFA and VFA using Synapse Vincent (Fujifilm Medical, Tokyo, Japan) [11]. The total cross-sectional SFA and VFA at the umbilicus level were calculated using Hounsfield unit thresholds of − 200 to − 50 for adipose tissue (Fig. 1). The cut-off values for SFA and VFA were 100 and 100 cm^2^, respectively [12, 13].

### Evaluation of surgical difficulty

A modified Japanese DSS (mDSS) for LDP was used to assess surgical difficulty (Table 1) [7]. The mDSS includes seven variables: type of surgery, malignancy, neoadjuvant therapy, pancreatic transection line, tumor close to the major vessel, tumor extension to the peripancreatic tissue, and left-sided portal hypertension and/or splenomegaly. The 12-level difficulty score was calculated and subcategorized into three levels: low (1–3), intermediate (4–6), and high (7–12).

**Table 1:** Modified difficulty scoring system for laparoscopic distal pancreatectomy

Parameter	Score
Type of operation	
Retroperitoneal dissection	+4
SPDP (Kimura)	+3
SPDP (Warshaw)	+1
Standard	+1
Malignancy	
Presence	+1
Absence	0
Neoadjuvant therapy	
Chemotherapy	+1
Radiotherapy	+1
Upfront	0
Pancreatic transection line	
Portal vein	+1
Pancreatic tail	0
Tumor close to major vessel	
Presence	+2
Absence	0
Tumor extension to peripancreatic tissue	
Presence	+1
Absence	0
Left sided portal hypertension	
Presence	+1
Absence	0
Difficulty index	Total score
Low	1–3
Intermediate	4–6
High	7–12

Difficulty parameters and index were evaluated according to the modified difficulty score system (7)

*SPDP* spleen-preserving distal pancreatectomy

### Surgical technique

Surgical protocols for RDP have been reported, including division of the gastrocolic ligament, dissection around the pancreas, division of the pancreas and splenic vessels, and mobilization of the pancreas and spleen using a medial approach [14]. In cases of pancreatic cancer, retroperitoneal dissection was performed using the radical antegrade modular pancreatosplenectomy technique [15]. In cases of benign tumors, the spleen-preserving technique with a splenic vessel-preserving approach (Kimura technique) or splenic vessel-sacrificing approach (Warshaw technique) was used [16]. During the study period, all procedures were performed and proctored by a single surgeon (KT) who received surgical training in robotic surgery in the Netherlands [17, 18].

### Statistical analysis

All statistical analyses were performed using JMP software version 11 (SAS Institute, Cary, NC, USA). Values are presented as medians (interquartile ranges [IQR]) for continuous variables and as proportions for categorical data. Initially, patient characteristics and difficulty indices were investigated. Subsequently, the outcomes were compared based on the difficulty index. Univariate and multivariate logistic regression analyses were performed to identify risk factors associated with prolonged operative time (> 217 min), which was defined using the median operative time [19]. Odds ratios (ORs) and 95% confidence intervals (CIs) were determined. Using the results of multivariate analyses, a simple DSS was generated, and the short-term outcomes of each group were compared. Lastly, internal validity was evaluated using the bootstrap method to assess the discriminative performance of the model [20]. The predictive validity of the model was assessed using the concordance index (C-index) of the calibration curve. In addition, internal validity was assessed using the tenfold cross-validation method to assess the discriminative performance of the model [21]. The area under the receiver operating characteristic curve was used to determine the predictive validity of the model.

## Results

### Study cohort

**Fig. 2:**
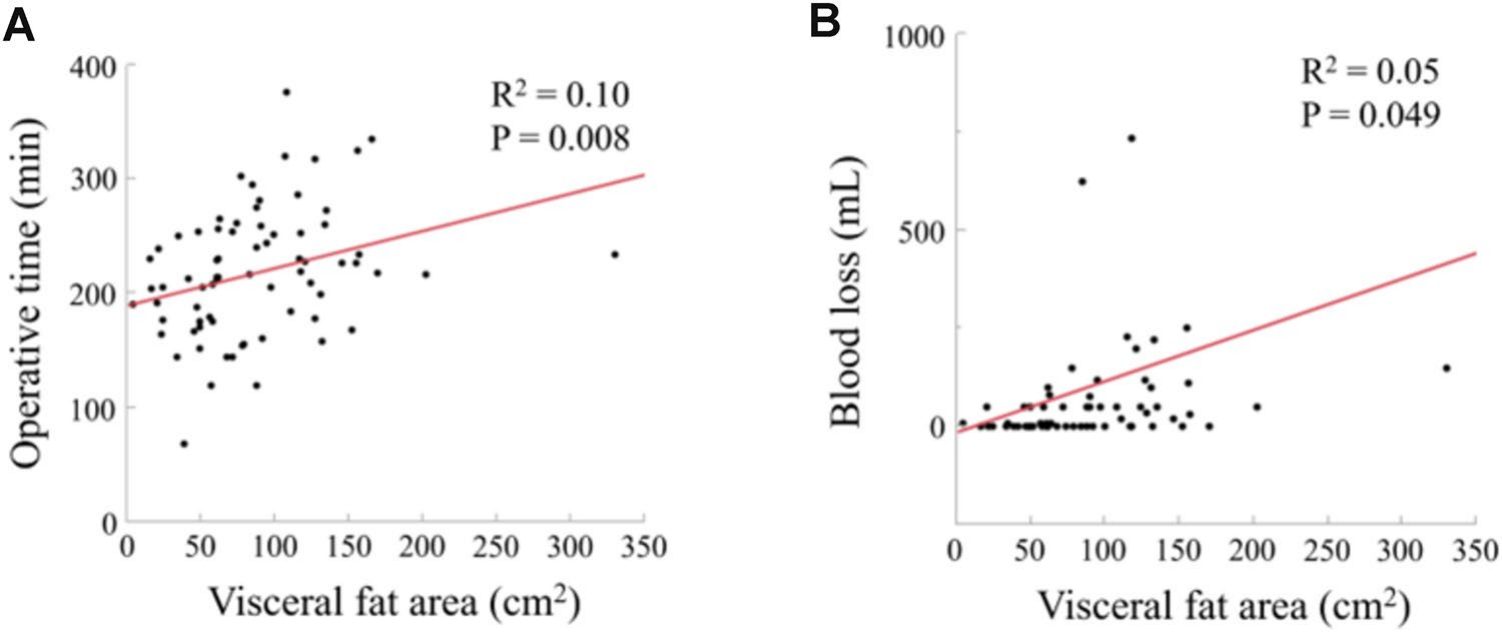
Relationship between visceral fat area (VFA) and operative outcomes. VFA was significantly associated with **A** operative time (r^2^ = 0.10, *P* = 0.008) and **B** estimated blood loss (r^2^ = 0.05, *P* = 0.049)

The characteristics of the 72 patients who underwent RDP are shown in Table 2. There were 31 men and 41 women with a median age of 71.5 years (IQR, 56–76). The most common primary disease was pancreatic cancer (n = 35, 48.6%), followed by neuroendocrine neoplasm (n = 12, 16.7%). Regarding body composition, the median SFA and VFA were 131 cm^2^ (IQR, 88–165) and 78 cm^2^ (IQR, 50–118), respectively. The association between the VFA and operative outcomes is shown in Fig. 2. VFA was significantly correlated with operative time (r^2^ = 0.10, P = 0.008) and estimated blood loss (r^2^ = 0.05, P = 0.049).

**Table 2:** Patient characteristics and difficulty parameters according to modified difficulty scoring system

Variables	n = 72
Age, years	71.5 (56–76)
Sex (men/women)	31 (43.1)/41 (56.9)
ASA (1–2/3–4)	45 (62.5)/27 (37.5)
Comorbidity	
Diabetes	18 (25.0)
Hypertension	30 (41.7)
Primary disease	
Pancreatic cancer	35 (48.6)
PNEN	12 (16.7)
IPMN	6 (8.3)
MCN	5 (6.9)
Others	14 (19.4)
Body composition	
Body mass index, kg/m^2^	22.8 (20.3–25.5)
Subcutaneous fat area, cm^2^	131 (88–165)
Visceral fat area, cm^2^	78 (50–118)
Difficulty parameter*	
Type of RDP	
Retroperitoneal dissection	28 (38.9)
SPDP (Kimura)	11 (15.3)
SPDP (Warshaw)	14 (19.4)
Standard	19 (26.3)
Malignancy	
Presence	36 (50.0)
Neoadjuvant therapy	
Chemotherapy	26 (36.1)
Radiotherapy	0 (0)
Upfront	46 (63.9)
Pancreatic transection line	
Portal vein	46 (63.9)
Pancreatic tail	26 (36.1)
Tumor close to major vessel	
Presence	17 (23.6)
Tumor extension to peripancreatic tissue	
Presence	5 (6.9)
Left sided portal hypertension	
Presence	1 (1.4)
Difficulty index*	
Low (1–3)	28 (38.9)
Intermediate (4–6)	25 (34.7)
High (7–12)	19 (26.4)

Values are reported as n (%), or median (interquartile range)

*ASA* American society of anesthesiologists, *PNEN* pancreatic neuroendocrine neoplasm, *IPMN* intraductal papillary mucinous neoplasm, *MCN* mucinous cystic neoplasm, *RDP* robotic distal pancreatectomy, *SPDP* spleen-preserving distal pancreatectomy

*Difficulty parameters and index were evaluated according to the modified difficulty score system (7)

### Difficulty index for RDP

The difficulty parameters evaluated using the mDSS are summarized in Table 2. The types of surgery included retroperitoneal dissection (n = 28, 38.9%), the Kimura technique (n = 11, 15.3%), the Warshaw technique (n = 14, 19.4%), and standard RDP (n = 19, 26.3%). The pancreatic transection line was located in the portal vein (n = 46, 63.9%) or pancreatic tail (n = 26, 36.1%). Ultimately, the patients were classified into three difficulty index groups: low (n = 28, 38.9%), intermediate (n = 25, 34.7%), and high (n = 19, 26.4%).

### Outcomes according to the difficulty index

The overall RDP outcomes are presented in Table 3. The overall median operative time and estimated blood loss were 217 min (IQR: 177–254) and 10 mL (IQR: 0–50), respectively. The incidences of mortality and major complications were 0 and 5.6%, respectively, with a median postoperative hospital stay of 9 days (IQR, 8–10). A textbook outcome was achieved in 84.7% of patients.

**Table 3:** Outcomes according to the difficulty index

Variables	Total (n = 72)	Low (n = 28)	Intermediate (n = 25)	High (n = 19)	*P* value
Operative factors					
Operative time, min	217 (177–254)	205 (167–230)	213 (177–237)	253 (214–275)	0.01
Estimated blood loss, mL	10 (0–50)	5 (0–73)	10 (0–50)	50 (0–100)	0.51
Conversion to open	0 (0)	0 (0)	0 (0)	0 (0)	–
Postoperative factors					
Mortality	0 (0)	0 (0)	0 (0)	0 (0)	–
Major complications (CDc ≥ 3)	4 (5.6)	1 (3.6)	2 (8.0)	1 (5.3)	0.78
POPF (≥ grade B)	7 (9.7)	3 (10.7)	3 (12.0)	1 (5.3)	0.71
PPH	0 (0)	0 (0)	0 (0)	0 (0)	–
Hospital stay, day	9 (8–10)	8 (7–10)	9 (8–11)	9 (9–10)	0.08
Readmission	8 (11.1)	5 (17.9)	2 (8.0)	1 (5.3)	0.34
Textbook outcome	61 (84.7)	22 (78.6)	22 (88.0)	17 (89.5)	0.51

Values are reported as n (%), or median (interquartile range)

*CDc* Clavien–Dindo classification (8), *POPF* postoperative pancreatic fistula, *PPH* postpancreatectomy hemorrhage

**Fig. 3:**
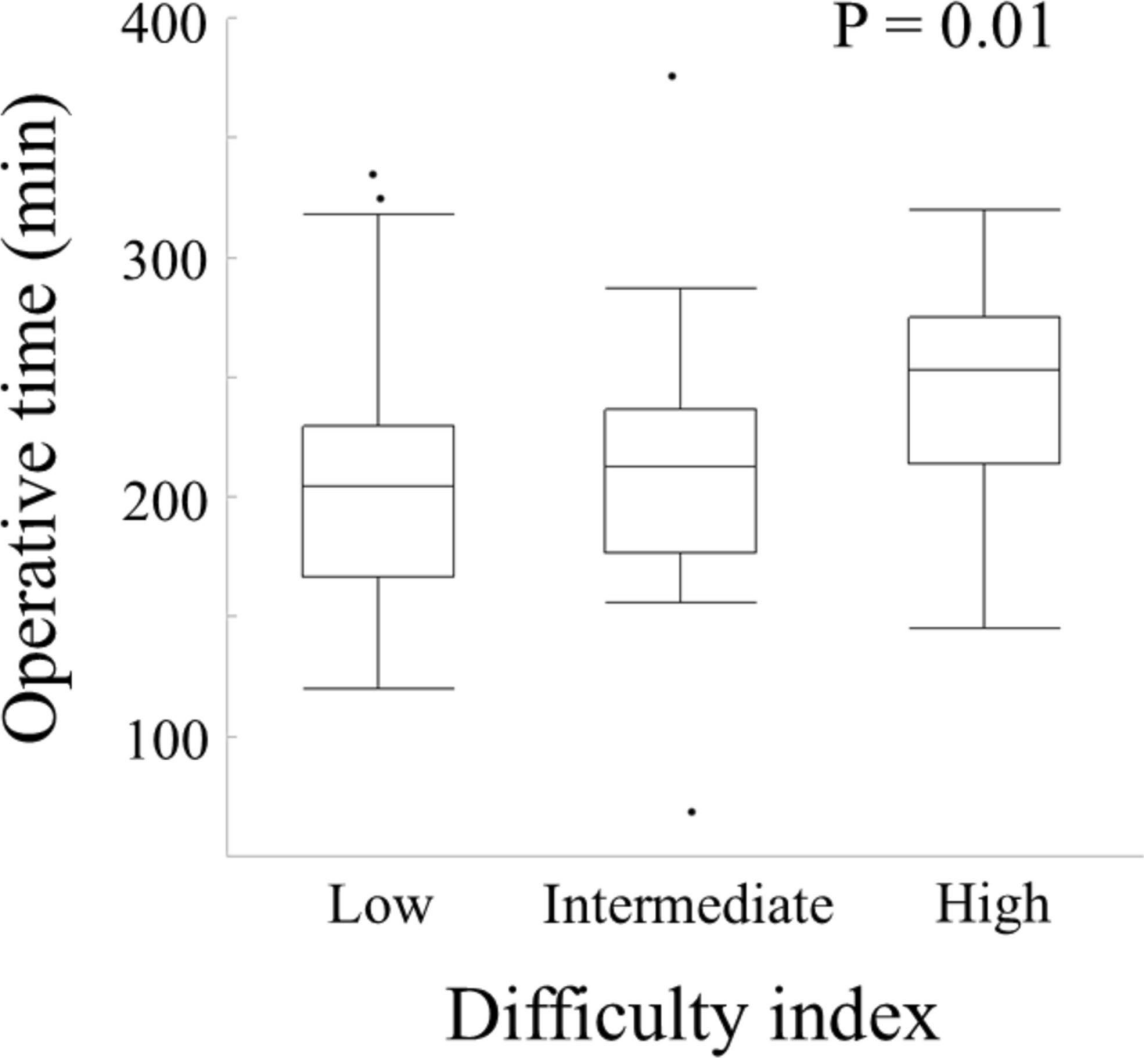
Relationship between operative time and the difficulty index. Operative time was the shortest in the low difficulty index group compared with the intermediate and high difficulty index groups. A significant difference was found between the three groups (P = 0.01)

The outcomes stratified by difficulty index are shown in Table 3. The operative time was significantly associated with the surgical index (P = 0.01; Fig. 3); however, no significant associations were found with the other outcomes, including estimated blood loss (*P* = 0.51), major complications (*P* = 0.78), length of hospital stay (*P* = 0.08), or a textbook outcome (*P* = 0.51).

### Risk factors for prolonged operative time and other endpoints

Table 4 shows the results of the univariate and multivariate analyses of the risk factors associated with prolonged operative time. In the univariate analysis, the following risk factors were significantly associated with prolonged operative time: body mass index, VFA, malignancy, and pancreatic resection line. Multivariate analyses revealed that VFA (≥ 100 cm^2^) (OR 5.03, 95% CI 1.32–22.4, *P* = 0.02), malignancy (OR 4.92, 95% CI 1.50–18.9, *P* = 0.01), and pancreatic resection on the portal vein (OR 4.14, 95% CI 1.24–15.9, *P* = 0.02) were significant risk factors for prolonged operative time.

**Table 4:** Univariate and multivariable analysis associated with prolonged operative time (> 217 min)

Variable	Univariate			Multivariable		
	OR	95% CI	*P* value	OR	95% CI	*P* value
Age, years						
≥ 70 (vs. < 70)	1.79	0.70–4.65	0.22			
Sex						
Men (vs. women)	1.55	0.61–4.02	0.36			
ASA						
3–4 (vs. 1–2)	1.56	0.60–4.14	0.36			
Body mass index, kg/m^2^						
≥ 25 (vs. < 25)	3.61	1.29–10.9	0.01	3.26	0.82–14.6	0.09
Subcutaneous fat area, cm^2^						
≥ 100 (vs. < 100)	2.13	0.81–5.80	0.13			
Visceral fat area, cm^2^						
≥ 100 (vs. < 100)	4.05	1.45–12.3	0.01	5.03	1.32–22.4	0.02
Type of operation						
Retroperitoneal dissection (vs. others)	2.23	0.86–6.01	0.10			
Malignancy						
Presence (vs. absence)	3.54	1.37–9.62	0.01	4.92	1.50–18.9	0.01
Neoadjuvant therapy						
Presence (vs. absence)	2.27	0.86–6.24	0.10			
Pancreatic resection line						
Portal vein (vs. pancreatic tail)	4.22	1.53–12.7	0.01	4.14	1.24–15.9	0.02
Tumor close to major vessel						
Presence (vs. absence)	0.92	0.30–2.76	0.88			
Tumor extension to peripancreatic tissue						
Presence (vs. absence)	4.65	0.65–93.4	0.13			

*OR* odds ratio, *CI* confidence interval, *ASA* American society of anesthesiologists

The univariate and multivariate analyses were performed to identify risk factors associated with other endpoints, including major complications, POPF, and failure to textbook outcome. As the incidences of major complications and POPF were relatively low, no significant factor was detected in the univariate analysis. In contrast, the multivariate analyses found that VFA (≥ 100 cm^2^) (OR 4.28, 95% CI 1.13–18.4, *P* = 0.03) was an independent factor associated with failure to textbook outcome (Table S1).

### Simple DSS for RDP

A simple scoring system was developed, with one point assigned to each significant risk factor (VFA ≥ 100 cm^2^, malignancy, and pancreatic transection line); the OR used was similar to that established in the multivariate analysis. According to the number of potential risk factors, the patients were divided into four groups: risk 0 (n = 12), risk 1 (n = 21), risk 2 (n = 32), and risk 3 (n = 7). As shown in Table 5, significant differences were observed between the groups in terms of operative time (*P* < 0.001), estimated blood loss (*P* = 0.005), and length of hospital stay (*P* = 0.02). No significant differences were found in textbook outcomes (*P* = 0.63).

**Table 5:** A simple difficulty grading system of robotic distal pancreatectomy

	Risk 0 (n = 12)	Risk 1 (n = 21)	Risk 2 (n = 32)	Risk 3 (n = 7)	*P* value
Operative time	174 (154–200)	205 (162–228)	237 (208–265)	261 (234–325)	< 0.001
Estimated blood loss	0 (0–8)	0 (0–50)	25 (0–50)	150 (50–734)	0.005
Mortality	0 (0)	0 (0)	0 (0)	0 (0)	–
Major complications (CDc ≥ 3)	0 (0)	2 (9.5)	2 (6.3)	0 (0)	0.44
POPF (≥ grade B)	0 (0)	4 (19.1)	3 (9.4)	0 (0)	0.13
PPH	0 (0)	0 (0)	0 (0)	0 (0)	–
Hospital stay	8 (7–8)	8 (7–10)	9 (9–11)	11 (8–14)	0.02
Readmission	1 (8.3)	2 (9.5)	3 (9.4)	2 (28.6)	0.60
Textbook outcome	11 (91.7)	17 (81.0)	28 (87.5)	5 (71.4)	0.63

Values are reported as n (%), or median (interquartile range)

*CDc* Clavien–Dindo classification (8), *POPF* postoperative pancreatic fistula, *PPH* postpancreatectomy hemorrhage

**Fig. 4:**
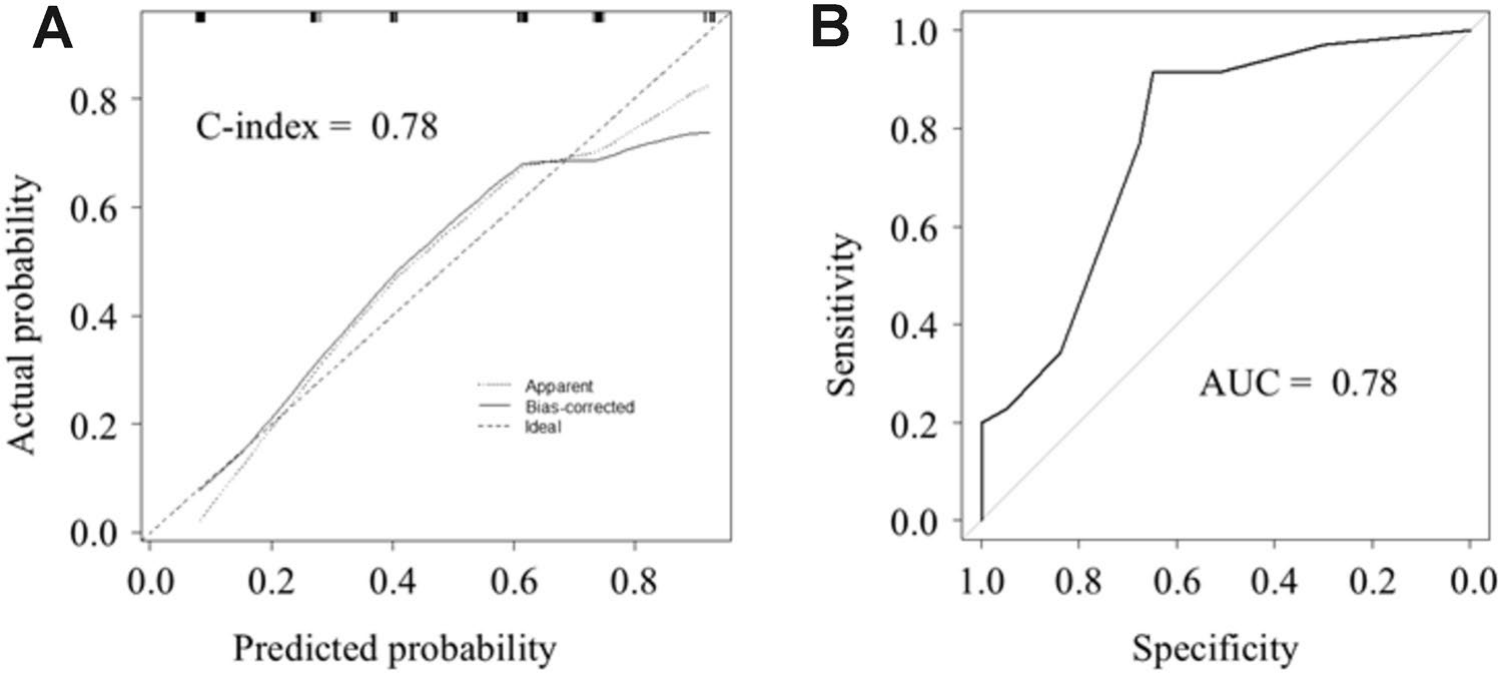
**A** The calibration plot of the model for predicting a prolonged operative time after robotic distal pancreatectomy. **B** The predictive performance of the model after based on tenfold cross-validation. The area under the curve (AUC) was 0.78

Calibration plots used to predict prolonged operative time using the bootstrap method are shown in Fig. 4A. The predicted probability of prolonged operative time moderately correlated with the actual probability, although it slightly underpredicted the incidence in the middle-risk group and overpredicted the incidence in the high-risk group. The C-index of the model was 0.78. Moreover, the adjusted C-index of the model, including confounders such as age and gender, was 0.76. Figure 4B showed the predictive performance of the model after performing tenfold cross-validation. The area under the curve was 0.78.

## Discussion

To the best of our knowledge, this is the first study to evaluate the surgical difficulty of RDP using the mDSS for LDP. The results validating the mDSS for RDP revealed that several risk factors from the mDSS were associated with a prolonged operative time during RDP. Moreover, the present study investigated the impact of VFA on surgical difficulty in RDP. We found that a larger VFA was significantly associated with a prolonged operative time in RDP. Our proposed simple scoring system, which includes VFA, may be helpful in estimating the surgical complexity of RDP.

Regarding overall outcomes, our results for RDP, including a textbook outcome, were relatively better than those of the international benchmark cutoffs [22, 23]. As we introduced RDP after the safe implementation of robotic pancreatoduodenectomy and had sufficient experience with robotic surgery [18], our learning curve for RDP may have been limited. Moreover, we have standardized surgical protocols for RDP, including strategies for approaching the splenic artery, performing retroperitoneal dissection, and spleen-preserving techniques [14–16].

The mDSS for LDP includes seven variables associated with surgical difficulty [7]. To date, these parameters have not been verified for application to RDP. Our results suggest that the difficulty index evaluated by the mDSS was significantly associated with operative time, which is a commonly used parameter for predicting surgical difficulty (Table 3) [19]. However, the difficulty index did not reflect other outcomes in this study, including estimated blood loss, postoperative complications, and hospital stay.

The multivariate analyses revealed that, of the seven difficulty parameters of the mDSS, only malignancy and the pancreatic resection line were independent risk factors associated with surgical difficulty (Table 4). We suggest that additional lymphadenectomy and dissection for malignant diseases prolong operative time. Moreover, pancreatic transection on the portal vein requires pancreatic tunneling by dissection between the pancreas and portal vein, resulting in a prolonged operative time. Our novel finding was the significant association between VFA and surgical difficulty in patients with RDP. Previous studies have reported the relationship between VFA and postoperative complications in gastrointestinal surgery [24–26]. Moreover, an association between visceral obesity and an increased risk of POPF has been demonstrated after pancreatectomy [27, 28]. Although VFA measurement may not be routine practice at most centers, it is simple, straightforward, and helpful in predicting the surgical difficulty of RDP. Therefore, we recommend the preoperative assessment of VFA in clinical practice.

Based on the multivariate analyses, we developed a simple DSS for RDP. Significant risk factors included longer operative time, greater blood loss, and longer hospital stay (Table 5). These results suggest that low-risk cases are more suitable for surgeons who are new to RDP or have experience with fewer than 20 cases [6]. Conversely, high-risk procedures should be undertaken by experienced surgeons. A recent study demonstrated that competency, proficiency, and mastery were achieved after 46, 63, and 73 RDP procedures, respectively; a longer and more complex process is required to master RDP [23]. Therefore, our simple DSS may be useful for patient selection for RDP.

This study had several limitations. This was a single-center retrospective study with a small sample size, potentially leading to selection bias and inadequate statistical power. Future research should include larger sample sizes and multicenter studies to validate these findings. We found that factors such as the VFA, malignancy, and pancreatic resection line were associated with the surgical difficulty of RDP. However, other confounding factors may also be associated with the VFA and surgical difficulty of RDP. Generally, variations among surgeons and their learning curves influence the surgical difficulty of RDP. Therefore, the generalizability of our findings may be limited. Moreover, external validation was not conducted to verify the applicability of VFA as an indicator for assessing the difficulty of RDP in different datasets or environments; consequently, the reliability and generalizability of this indicator may be limited. Considering the differences in body composition among countries worldwide, our findings should be confirmed in Western cohorts.

In conclusion, this study investigated the association between the difficulty index, stratified by the mDSS, and outcomes after RDP. Moreover, the effect of VFA on outcomes after RDP was analyzed. Assessment of VFA may be helpful in predicting the surgical difficulty of RDP. Further studies are required to validate the effectiveness of the proposed simple DSS for RDP.

## Supplementary Information

Below is the link to the electronic supplementary material.

Supplementary file1 (DOCX 21 KB)
