# Supplementary material for: Impact of visceral fat area on surgical difficulty during robotic distal pancreatectomy (TAKUMI-2)
Source: Surg Endosc. 2025 Apr 4;39(5):3137–45. doi: 10.1007/s00464-025-11696-3 (PMC12041091; doi:10.1007/s00464-025-11696-3)
Supplement: Supplementary file 1 — Supplementary file1 (DOCX 21 KB) [file 464_2025_11696_MOESM1_ESM.docx]

**Table S1** Univariate and multivariable analysis associated with failure to textbook outcome

| Variable | Univariate | | |  | Multivariable | | |
| --- | --- | --- | --- | --- | --- | --- | --- |
|  | OR | 95% CI | P value |  | OR | 95% CI | P value |
| Age, years |  |  |  |  |  |  |  |
| ≥70 (vs. <70) | 0.62 | 0.16–2.27 | 0.47 |  |  |  |  |
| Sex |  |  |  |  |  |  |  |
| Men (vs. women) | 1.73 | 0.47–6.60 | 0.41 |  |  |  |  |
| ASA |  |  |  |  |  |  |  |
| 3–4 (vs. 1–2) | 1.48 | 0.39–5.46 | 0.56 |  |  |  |  |
| Body mass index, kg/m^2^ |  |  |  |  |  |  |  |
| ≥25 (vs. <25) | 1.99 | 0.52–7.45 | 0.31 |  |  |  |  |
| Subcutaneous fat area, cm^2^ |  |  |  |  |  |  |  |
| ≥100 (vs. <100) | 1.73 | 0.45–8.49 | 0.44 |  |  |  |  |
| Visceral fat area, cm^2^ |  |  |  |  |  |  |  |
| ≥100 (vs. <100) | 4.53 | 1.21–19.2 | 0.02 |  | 4.28 | 1.13–18.4 | 0.03 |
| Type of operation |  |  |  |  |  |  |  |
| Retroperitoneal dissection (vs. others) | 0.30 | 0.04–1.28 | 0.11 |  |  |  |  |
| Malignancy |  |  |  |  |  |  |  |
| Presence (vs. absence) | 0.52 | 0.12–1.90 | 0.32 |  |  |  |  |
| Neoadjuvant therapy |  |  |  |  |  |  |  |
| Presence (vs. absence) | 0.34 | 0.05–1.47 | 0.16 |  | 0.38 | 0.05–1.72 | 0.22 |
| Pancreatic resection line |  |  |  |  |  |  |  |
| Portal vein (vs. pancreatic tail) | 0.99 | 0.27–4.11 | 0.98 |  |  |  |  |
| Tumor close to major vessel |  |  |  |  |  |  |  |
| Presence (vs. absence) | 0.68 | 0.10–3.03 | 0.64 |  |  |  |  |
| Tumor extension to peripancreatic tissue |  |  |  |  |  |  |  |
| Presence (vs. absence) |  |  |  |  |  |  |  |

OR, odds ratio; CI, confidence interval; ASA, American Society of Anesthesiologists.
